# History of a Case of Severe Affection from the Bite of a Viper

**Published:** 1820-03

**Authors:** 


FOR THE LONDON MEDICAL AND PHYSICAL JOURNAL.

### *History of a Case of severe Affection from the Bite of a Viper.*

By Dr. Meischner.

A PEASANT boy, thirteen years of age, gathering wild berries, was bit by a viper *(coluber berus)* at the second joint of the third finger of the right hand, at ten o'clock in the morning of the 23d of July, 1818. The bitten part soon became swelled, hot, and the finger contracted; and this contraction and swelling soon affected the arm, the latter extending above the elbow. It was eight o'clock on the same day when I first saw the patient: a tight bandage had been applied to the hand and arm. The wound was very small, not deep; indeed, like that from the prick of a pin; there was not the slightest mark of inflammation around it. The whole of the right arm was much swelled, especially about the joints; the skin was of a deep-red colour; the finger cold, but not without feeling. The respiration was short and oppressed, and the patient complained of flying pains in the chest; the head felt heavy; and he had great thirst. The pulse in the healthy arm was quick, strong, and full: on the contrary, that of the wounded arm was small and low. The skin generally was dry. The bandage was wholly removed; the wound scarified and dressed with a digestive salve containing powdered cantharides; and the arm was fomented with olive-oil, and directions given for it to be frequently repeated. I prescribed a grain of powder of belladonna to be taken inwardly every two hours. The night was passed in a very restless state, and with great disturbance and difficulty of respiration.

On the ensuing day, the swelling of the arm and adjacent part of the integuments of the chest was somewhat lessened, as well as the redness of the skin; but the boy complained of great pain in the belly, and the abdomen was contracted inwards; but the evacuation of the fæces and urine was not disturbed. After four doses of the belladonna had been taken, it became necessary to discontinue its use; for it had produced vertigo, sickness at the stomach, dilatation of the pupils, &c. I applied six cupping-glasses, with the scarificator, to the right arm, and gave inwardly a concentrated decoction of *Virginian serpentary*, with the liquor of acetated ammonia, a spoonful at a time, frequently. The dressing and fomentation were continued. The ensuing night was passed more quietly; the stiches in the chest, and oppression of breathing, were much less severe.

On the 25th, the swelling and redness of the arm had much diminished; the bitten part discharged a little matter; the hand was naturally warm, the chest tolerably free. The abdomen was, however, still drawn inwards, and the right side of the scrotum contracted, and of a yellowish-green colour, changing afterwards to a brownish hue; all the rest of the right side was of a dull-brown colour. Sleep and appetite had increased; the pulse was nearly natural, but the skin still dry. On the evening of this day, a gentle sweat broke out, which became more abundant during the night, and gradually carried off the remainder of the swelling of the arm. The skin by degrees resumed its natural colour, presenting various hues in succession, namely, yellow, greenish, blue, and then a deep-brown: this was particularly observable in the scrotum. Under the use of the remedies above mentioned, which were still continued for some time hence, the boy at length fully recovered his health.

A similar case was also successfully treated by me in the same way. Two other children, who were in the same summer* bitten by vipers, lost their lives: in these no professional assistance was resorted to.

*Hartenstein, in Erzgebirge.*

* The summer of 1818 was remarkably hot and dry in Germany, as well as in the rest of Europe; and in such a season vipers are always very abundant, and are more disposed to attack persons than they are in general.

